# Microfluidics as efficient technology for the isolation and characterization of stem cells

**DOI:** 10.17179/excli2020-3028

**Published:** 2021-02-22

**Authors:** Afsoon Aghlmandi, Aylin Nikshad, Reza Safaralizadeh, Majid Ebrahimi Warkiani, Leili Aghebati-Maleki, Mehdi Yousefi

**Affiliations:** 1 Department of Animal Biology, Faculty of Natural Science, University of Tabriz, Tabriz, Iran; 2 The School of Biomedical Engineering, University of Technology Sydney, Sydney, NSW, Australia; 3 Immunology Research Center, Tabriz University of Medical Science, Tabriz, Iran; 4 Stem Cell Research Center, Tabriz University of Medical Science, Tabriz, Iran

**Keywords:** stem cell, cell isolation, microfluidic, FACS, MACS

## Abstract

The recent years have been passed with significant progressions in the utilization of microfluidic technologies for cellular investigations. The aim of microfluidics is to mimic small-scale body environment with features like optical transparency. Microfluidics can screen and monitor different cell types during culture and study cell function in response to stimuli in a fully controlled environment. No matter how the microfluidic environment is similar to *in vivo* environment, it is not possible to fully investigate stem cells behavior in response to stimuli during cell proliferation and differentiation. Researchers have used stem cells in different fields from fundamental researches to clinical applications. Many cells in the body possess particular functions, but stem cells do not have a specific task and can turn into almost any type of cells. Stem cells are undifferentiated cells with the ability of changing into specific cells that can be essential for the body. Researchers and physicians are interested in stem cells to use them in testing the function of the body's systems and solving their complications. This review discusses the recent advances in utilizing microfluidic techniques for the analysis of stem cells, and mentions the advantages and disadvantages of using microfluidic technology for stem cell research.

## Introduction

It is a well-recognized fact that cells are essential elements of life with the ability to communicate with each other and the components of the extracellular environment. So far, great bulk of studies have been performed to investigate the intrinsic potency of stem cells in terms of properties such as self-renewal and proliferation. However, there are many limitations that stem cell research has faced which complicate our perception of this scientific field. One of the most significant considerations in fundamental and functional stem cell research is the effective reproduction and isolation of pure cells, which can be used to examine the heterogeneity of stem cell populations (Sart et al., 2014[103]). Stem cells play important roles in living organisms. In blastocyst stage (five or six days after fertilization), the inner layer cells turn into the entire body of the organism, including a variety of specialized cell types and organs such as heart, lung, skin, sperm, egg and other tissues. In some adult tissues such as bone marrow, muscle, and brain, there are distinct populations of stem cells, called adult stem cells, which are responsible for replacing cells being lost through normal wear and tear, injury, and diseases. 

Over the past decades, stem cells have been successfully applied to regenerate different types of tissues and organs (Aghebati-Maleki et al., 2019[1]; Pourakbari et al., 2020[92]). The importance of stem cell research in the clinical context has made it necessary to develop effective techniques for the isolation and evaluation of these cells. Although there are many ways to commercially isolate available and extensively-used stem cells, the efficiency and specificity of these methods are still insufficient. Separating stem cells is a complicated task because these cells are sensitive to the environmental stimuli as well as the interferences between different cell types. Current isolation techniques depend on the recognition of surface antigens specifically expressed on stem cells. These techniques include surface immunolabeling, which is based on detection by flow cytometry and magnetic sorting. Due to the limitations of these techniques, finding a method needless of labeling is very important (Pourakbari et al., 2020[93]). The microfluidic approach of screening and manipulating cells is a newly developed but extensively used technology which does not need cells labeling. The use of this technology has led to the separation of stem cells without labeling, leaving the shape and specific cellular properties, such as electrical properties, unchanged. In the current review, we have discussed stem cells and different methods used for their isolation. We have also explained microfluidic method and the advantages of this technology in stem cell isolation.

## Classification of Stem Cells

Stem cells are undifferentiated cells which can be differentiated into functional cells (Bongso and Fong 2009[6]; Larijani et al., 2012[60]). These cells are distinguished from other cell types by two important characteristics. At first, they are undifferentiated cells capable of self-renewal through cell division. This renewal can be happened after long period of inactivity (Smith, 2005[113]; Hosseinkhani and Hosseinkhani, 2009[44]; Larijani et al., 2012[60]; Sobhani et al., 2017[115]). Second, under specified physiological or laboratorial circumstances, they can be induced to become specific cells forming different tissues and organs (Kimmelman et al., 2016[56]). To date, researchers have mainly focused on two types of animals and humans-derived stem cells (Sart et al., 2014[103]). In 2006, stem cell research experienced a significant discovery when researchers reached to conditions that would permit some specialized adult cells to be "reprogrammed" genetically and turn into a stem cell-like state. This novel type of stem cells are called Induced Pluripotent Stem Cells (IPSCs) (Desai et al., 2015[18]).

Embryonic stem cells are originated from cells existing in the embryo with just a few days of life. In humans, mice and other mammals, the embryo is a ball of nearly 100 cells at this phase. These cells, which are called blastocyst, have two parts: an external layer of cells and trophectoderm or trophoblast which gives rise to the placenta that protects the embryo when it grows inside the uterus. An internal aggregation of cells, the Inner Cell Mass (ICM), is a ball of 10-20 cells. These cells are undifferentiated and their function has yet to be determined. The ICM is divided and its cells are differentiated to generate all the cells needed to form the entire animal. Some of the cells in ICM are pluripotent, meaning that they can produce different cell types of the body (Weissman, 2002[136]; Nandedkar and Narkar, 2003[77]; Li and Xie, 2005[65]; Fu et al., 2011[29]; Kutty and Kumar, 2012[59]).

According to their differentiation capacity, stem cells can be divided into several groups (4): totipotent, pluripotent, multipotent, and unipotent cells. Totipotent cells exist in the 4- to 8-cell stage of embryo and can create all body tissues, amniotic membrane (the pleura), and chorionic membrane (pericardium). Totipotent cells can be isolated from 4- or 5-day-old embryos. These embryos are derived from In Vitro Fertilization (IVF) oocytes which are cultured under specific circumstance. For the same reason, totipotent cells are known as embryonic stem cells (Bongso and Fong, 2009[6]; Hosseinkhani and Hosseinkhani, 2009[44]; Sobhani et al., 2017[115]). Pluripotent cells are another group of stem cells that can produce cells of different tissues belonging to ectoderm, mesoderm, and endoderm referring to the outer, middle, and inner layers of the embryo, respectively. Ectodermal stem cells are precursors of skin cells as well nervous system cells. Mesodermal cells are precursors of nerve, muscle, fat, connective tissue, blood, and renal tubular cells and endodermal cells are precursors of pancreatic, thyroid, and lung cells. In adults, a type of highly potent mesodermal stem cells is found in tooth pulp and some adipose tissues. Some of these cells (embryonic hematopoietic stem cells) can be obtained from the blood left in the umbilical cord after childbirth. An important characteristic of umbilical cord blood is the presence of a high number of immature lymphocytes. Therefore, once it is transplanted to the bone marrow of patient, besides providing him/her with new blood cells, one can expect high rate of graft success. Since embryonic blood stem cells are pluripotent and capable of differentiating into all body tissues, their isolation from the umbilical cord opens up new horizons in the treatment of different diseases in the future (Smith, 2005[113]; Jaenisch and Young, 2008[47]; Kutty and Kumar, 2012[59]; Larijani et al., 2012[60]). Multipotent cells have lower potency than pluripotent cells and can produce different cells of specific tissues. Bone marrow stem cells are instances of multi potency. These cells can give rise to different cell types of blood tissue including red blood cells, white blood cells (leukocytes), and platelets. Unipotent stem cells are found in adult tissues and can be differentiated along a single lineage. B-lymphocytes are an example of unipotent stem cells, which can only produce plasmocytes (Weissman, 2002[136]; Nandedkar and Narkar, 2003[77]; Li and Xie, 2005[65]; Bunnell et al., 2008[8]; Fu et al., 2011[29]; Yoshida and Yamanaka, 2011[148]). Totipotent cells are another class of stem cells that can be differentiated into all cell types existing in the body. The distinction between pluripotent and multipotent stem cells is that the pluripotent cells can be differentiated into approximately all cell types, but multipotent stem cells only can be differentiated into cell types present in the same family (Ulloa-Montoya et al., 2005[129]; Mitalipov and Wolf 2009[75]).

**Figure 1:**
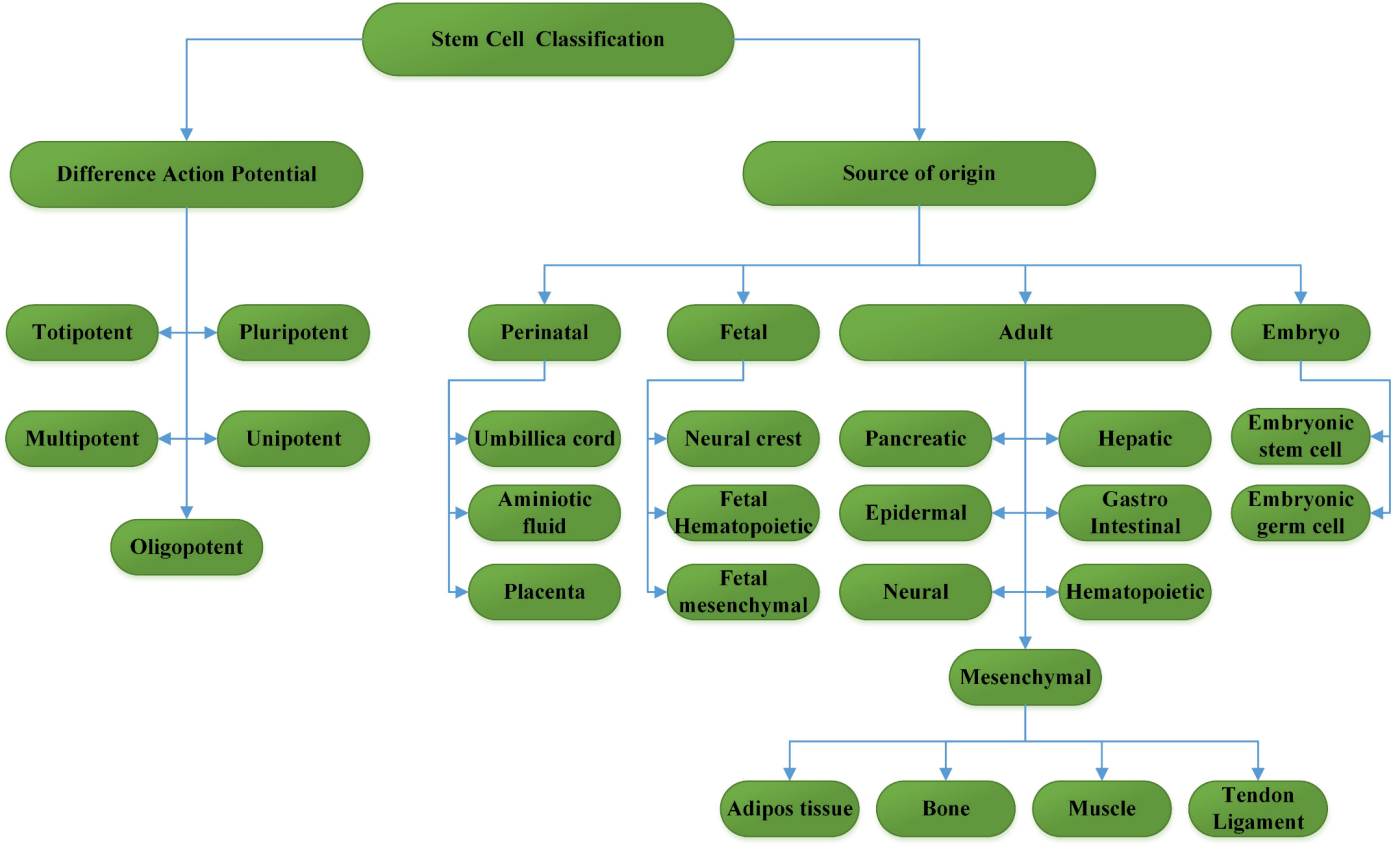
Stem cell classification with two features. Stem cells give rise to generate differentiated cells via a committed population

In general, stem cells are divided into two main groups: embryonic stem cells and adult stem cells (Figure 1(Fig. 1)).

### Embryonic Stem Cells (ESCs)

Embryonic Stem Cells (ESCs) are isolated from ICM or pluripotent blastocytes and can produce all derivatives of the embryonic layer except the extracellular tissue (Wu et al., 2011[140]). Because of their capacity to generate all cell types ESCs became a valuable tool in drug development and cell therapy (Rohwedel et al., 2001[101]). For example, cells isolated from ESCs are used in drug screening and toxicology. In some genetic diseases, these cells can be also labeled and traced (Rohwedel et al., 2001[101]; Menendez et al., 2006[74]; Di Giorgio et al., 2007[21]).

### Adult stem cells

Tissue-specific stem cells, also known as somatic or adult stem cells, are more specialized than embryonic stem cells. They can produce different types of cells in the tissue or organ in which they are found. Hematopoietic Stem Cells (HSCs), Neural Stem Cells (NSCs), and Mesenchymal Stem Cells (MSCs) are the instances of adult stem cells. NSCs are multipotent cells which can form different mature cells in the tissue in which they exist, but these cells cannot completely regenerate the whole organism. NSCs are self-renewable cells and produce three types of cells present in the nervous system including: neurons, astrocytes and oligodendrocytes (Gregg et al., 2002[35]). NSCs can be widely used in the treatment of neurodegenerative diseases such as Parkinson's and Alzheimer's diseases, but are not similar to stem cells when re-transplanted (Doetsch et al., 2002[22]; Marshall et al., 2006[72]). 

**Figure 2:**
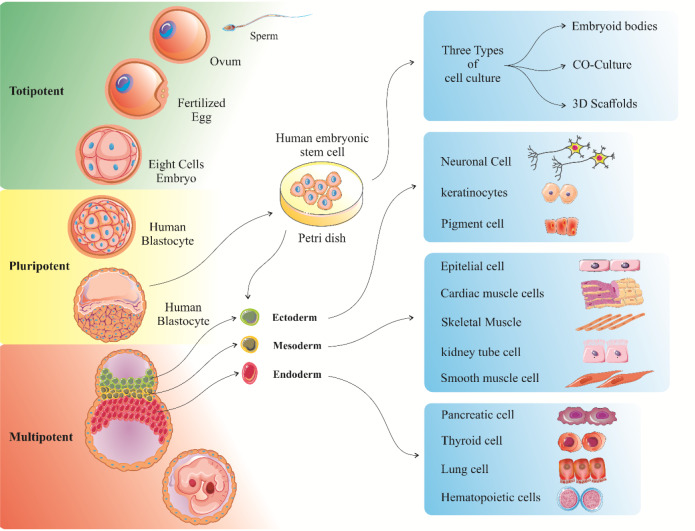
Schematic illustration of stem cells that can be isolated from human body and are able to differentiate into various cell types

Adult stem cells can be found in the bone marrow, peripheral blood, teeth, liver, ovary, heart and epithelium (in 't Anker et al., 2003[46]; Wu et al., 2009[139]). HSCs exist in the bone marrow and can generate red/ white blood cells, and platelets. However, HSCs cannot generate liver, lung or brain cells. Similarly, stem cells in other tissues and organs cannot produce red/white blood cells, and platelets. HSCs are self-renewable cells that can make different blood cells (Dhot et al., 2003[20]). They are isolated from the umbilical cord and can be considered as a potent cellular source for transplant purposes because they are capable of reducing the incompatibility of Human Leukocyte Antigens (HLAs) in donors (Wu et al., 2011[140]). MSCs can be isolated from Stromal Cells (SCs). SCs are connective tissue cells that create a supportive structure for the functional cells of a certain tissue (Pittenger, 1999[89]). The first MSCs were found in bone marrow and were shown to be able to produce bone, cartilage, and fat cells. Since then, MSCs have been separated from other tissues such as fat and umbilical cord blood (Pittenger, 1999[89]). Various MSCs are thought to possess stem cells and even immunomodulatory properties and have been examined as a therapeutic approach in many disorders. However, there is little evidence indicating their therapeutic efficiency. Up to now, researchers have not fully realized that these cells are actually stem cells and what cell types they can generate. There is general agreement MSCs are not similar, and their characteristics depend on the part of the body they are originated and how they are isolated and grown (Pountos et al., 2006[91]). MSCs isolated from umbilical cord (Kassem et al., 2008[52]), placenta (Lee et al., 2004[63]), and amniotic fluid (Yang et al., 2018[145]) are able to be differentiated into cells derived from all three primary germ layers of embryo (ectoderm, mesoderm and endoderm). Some tissues and organs within the body such as skin, blood, and lining of the gut contain a small population of tissue-specific stem cells whose function is to replace lost cells from these tissues. Tissue-specific stem cells are probably hard to find in the human body and have not self-renewal properties in culture easily like embryonic stem cells. However, our general knowledge of normal development, aging, and molecular aspects of injury and diseases has been increased by the study of these types of cells (Weissman, 2002[136]; Nandedkar and Narkar, 2003[77]; Li and Xie, 2005[65]; Smith, 2005[114]; Jaenisch and Young, 2008[47]; Bongso and Fong, 2009[6]; Fu et al., 2011; Niibe et al., 2011[80]; Larijani et al., 2012[60]; Desai et al., 2015[18]; Kimmelman et al., 2016[56]). Induced pluripotent stem (IPS) cells are lab-engineered cells that result from the conversion of tissue-specific cells, such as skin cells, into cells that behave like embryonic stem cells. IPS cells are key tools in helping researchers to have further knowledge on disease normal development as well as its onset and progression. These cells are also efficient in testing and developing new drugs and therapies. Despite that IPS cells have many common characteristics with embryonic stem cells (e.g. the ability to produce all cell types in the body) they possess different properties with embryonic stem cells. Researchers are trying to explore what these differences are and what is their biological meaning. For example, the first IPS cells were produced using viruses to insert extra-copies of genes into tissue-specific cells. Now, researchers are trying different approaches to create IPS cells to use them as a source of cells or tissues for medical treatments (Figure 2(Fig. 2)) (Shi et al., 2017[107]).

## Stem Cell Cultivation

Limitations in animal models have made researchers to look for alternative ways in the study of human diseases and biology. Over the recent years, the culture of immortal cells, such as embryonic stem cells and pluripotent cells in bio-reactors has opened new horizons in front of regenerative medicine and tissue engineering. In the following section, we presented an overview of the importance of new methods for the isolation of stem cells in both research and biomedical applications (Tandon et al., 2013[121]).

## Cell Separation Methods

Understanding how to isolate stem cells in the laboratory (Shamblott et al., 1998[105]; Thomson, 1998[125]) has made stem cell-based development of therapeutic approaches to greatly progress (Obradovic et al., 2008[81]; Fox et al., 2014[28]). The most commonly used stem cells for clinical applications are bone marrow and umbilical cord-derived stem cells applied on patients undergoing chemotherapy (Barker et al., 2003[3]; Obradovic et al., 2008[81]; Fox et al., 2014[28]). Nowadays, stem cells are considered as a therapeutic approach for a variety of diseases such as lung cancer (Gautam et al., 2015[31]; Chen and Hou, 2016[12]), neurological disorders (Kim et al., 2015[55]; Laroni et al., 2015[61]; Goldman, 2016[32]), heart (Jastrzebska et al., 2016[48]; Mathur et al., 2016[73]; Talkhabi et al., 2016[120]) and kidney pathologies (Suzuki, 2016[118]; Mansournia et al., 2017[71]) as well as neurodegenerative disorders such as Parkinson's, Alzheimer's, and Multiple Sclerosis (MS).

**Figure 3:**
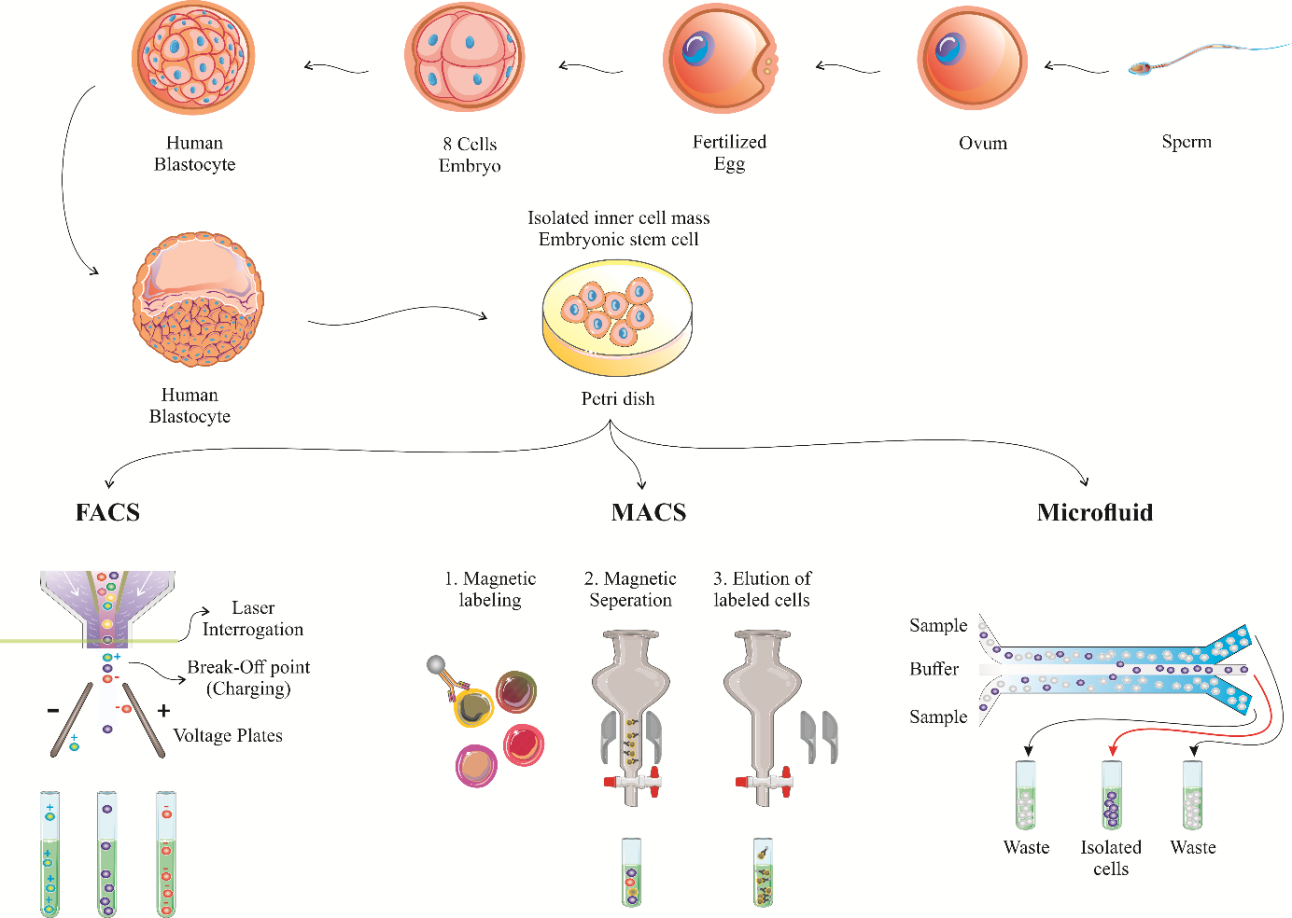
The procedure of stem cell isolation. Schematic diagram for stem cell sorting using FACS, MACS and microfluidics. FACS: fluorescence-activated cell sorting; MACS: magnetic-activated cell sorting

In order to use stem cells as transplantation therapeutic approach, they must have high efficiency and purity. Purity is the ratio of the isolated target cell to the whole cells. Here, the first step is to isolate, cultivate, and enrich stem cells (Schwartz et al., 2012[104]). The methods identified so far are divided into two main categories. The first group is based on evaluating physical parameters such as size and density. The second group is based on the affinity of chemical, electrical, and magnetic connections (Radisic et al., 2006[97]). The mechanism of the first category is based on the fact that stem cells of the same tissue have certain sizes and densities. The simplest example of this type is the density-gradient centrifugation method that creates a density gradient in the test tube and the soles, after being exposed to the escaping force from the center, are layered along the density gradient (Brakke, 1951[7]). Field Flow Fractionation (FFF) is another approach needless of labeling. In this technique, cells are collected at different time intervals according to size and morphology. Therefore, cells are separated only according to their physical parameters (Pappas and Wang, 2007[83]; Gothard et al., 2011[33]). Di Electrophoresis (DEP) is another labeling-free approach in which cells are arranged according to their electrical properties. The cell is a polar body and when placed in a non-uniform electric field, its direction is dependent on the electrical charge on its surface and its nucleic acid content (Gothard et al., 2011[33]). The main limitation in these methods is that the size and density of stem and non-stem cells are not completely different. Sometimes, these properties are very similar between stem and non-stem cells. Therefore, during separation, stem cells may become contaminated with non-stem ones. To solve this, label-dependent separation methods are used to characterize stem cell-specific markers. Here, the famous method is Fluorescence-Activated Cell Sorting (FACS). In this technique, fluorescent dyes bind to the mixture of cells and the cells are then separated according to the fluorescent light they produce (Thiel et al., 1998[124]; Putnam et al., 2003[95]). In this approach, the output is 10^7^ cells per hour with the efficiency of 90 %. However, cells are exposed to high pressure which may be destructive (Fong et al., 2009[27]; Chapman et al., 2013[11]). An alternative to FACS is Magnetic-Activated Cell Sorting (MACS). In MACS, a magnetically conjugated antibody is used to isolate the magnets. The speed of separation of this technique is about 10^11^ cells per hour. However, in both FACS and MACS, the percentage of stem cell purity is at risk, and the fluorescent and magnetic markers might act as contaminants that interfere with cell proliferation and differentiation (Fong et al., 2009[27]; Chapman et al., 2013[11]). Also, the high number of surface markers and their dependence on the expression of various factors such as growth factors, patient status and culture medium makes the utilization of these separation techniques a difficult task. One of the main objectives of stem cell isolation is to use them in transplantation and regenerative medicine. These applications need a high purity of cells. In addition, FACS-based methods are expensive and time-consuming. Thus, it has made the researchers to develop label-free methods. An important methodology is microfluidics, which uses physical parameters such as shape, size, adhesion, and electrical cell identifiers for cell separation (Figure 3(Fig. 3)) (Zhu and Murthy, 2013[152]; Reinhardt et al., 2015[98]; Rodrigues et al., 2015[100]).

## Microfluidics

Microfluidics were initially introduced about two decades ago (Kim et al., 2006[54]; Zhu et al., 2017[153]) and have been used to demonstrate the differentiation of stem cells exposed to different biochemical signals, such as cytokines and growth factors in a controlled medium (Young and Beebe, 2010[149]; Gupta et al., 2011[37]). There are two types of microfluidic techniques in cell separation: active and inactive microfluidics that is based on physical parameters such as electrical, magnetic, optical, and acoustic forces (Shields 4^th^ et al., 2015[108]; Lee et al., 2016[62]). Both methods work according to the biophysical and biochemical intercellular differences (Jubery et al., 2014[49]; Cemažar et al., 2016[9]). Microfluidic systems are highly advanced with many applications and utilize 3-Dimensional (3D) cell culture systems (Dvir et al., 2011[23]). After placing the samples in device, their performance can be filmed entirely. Devices were initially utilized to interact between cells, but later on, concentration and flow gradients were also utilized in devices. Both of these properties can be used in combination in devices. Sometimes, by placing a layer on devices and creating hypoxic conditions, the amount of cellular oxygen can be measured. When compared to the conventional plates, this system offers some advantages including the ability to control the spatial distribution of physical and chemical signals from cell surface, the ability to do cell analysis, and simultaneously performing several ways. In addition, these devices can solve the challenges of conventional cell culture methods and provide an opportunity for high-resolution imaging (Young and Beebe, 2010[149]; Han et al., 2012[39]; Li et al., 2014[67]). Microfluidic technology enables fluid manipulation in the range of the micro- to the pico-liters levels (Whitesides 2006[137]). Microfluidics use interdisciplinary knowledge of biotechnology, physics, and engineering (Karimi et al., 2016[51]). These devices provide a suitable microenvironment for cells and tissues. In other words, researchers can control the microenvironment surrounding cells with tiny tools (Wang et al., 2014[135]). Microfluidic devices help biologists to protect and analyze cell growth in a controlled environment (Wang et al., 2014[132]). The merge of microfluidic systems with stem cell technology can create a more suitable condition for stem cell culture when compared to other cell types. Microfluidic cell culture has several advantages mainly including the production of a uniform population, a combination of multilayer controlled release and flow signaling, and the capacity to co-culture in a 3D medium (Kassem et al., 2008[52]). Some important factors in the world of physics, such as gravity and inertia, are used in microfluidic experiments. Properties such as diffusion, surface tension, and viscosity at the micrometer scale function is very similar to what happens in the body (Ziebis et al., 1996[154]). Microfluidic method is a way to manipulate liquid droplets and is an advanced technology capable of controlling surface properties spatially (Gascoyne et al., 2004[30]). In this technique, a thin border layer separates two distinct phases of the material, which can be solid, liquid or gas with different properties (Pollack et al., 2000[90]). Shrinking a micrometer-scale system increases the surface area to volume ratio. As a result, there is a relatively higher transmission relation and it requires less mass and energy to reach the final state. Therefore, due to the reduced size of the system, homogenization heat transfer is done well. Smaller fluid behavior is increasingly affected by viscosity rather than inertia (Koschmieder, 1993[58]; Bird et al., 2001[4]). A simple microfluidic device used to create and manipulate droplets is ‘T-junction’. The two flows of unmixable liquids are forced to the T-shaped channel geometry to be combined in a way that one liquid forms droplets to be dispersed in the other (Thorsen et al., 2001[126]). The droplet-forming stage can be selected by regulating the hydrophobicity of device walls at the junction and the partial flow level of liquids (Okushima et al., 2004[82]). The use of T-junctions in series with surrogate surface wettabilities produces monodisperse double emulsions that makes benefits for encapsulation applications or extractions across the thin layer separating the internal droplets and the continuous phase (Utada, 2005[130]). Microscopic fluid behavior can differ from macrofluidic behaviors in terms of properties such as surface tension, energy dissipation, and the onset of fluid resistance to the system. Microfluidics studies can show how these behaviors change and how they can work (Terry et al., 1979[123]; Qiao, 2006[96]; Smistrup et al., 2008[112]; Kirby, 2010[57]). At smaller scales (channel sizes from about 100 nanometers to 500 micrometers) the rules beyond our rational interpretation are applicable. In other words, the Reynolds unit, which compares the effect of momentum or fluid momentum with the effect of viscosity, can be greatly reduced. A key result is that the same fluids flows in their traditional sense do not necessarily mix together because the flow is relaxed rather than perturbed; the molecular relation between them should be by distribution (Tabeling, 2005[119]). In this manner, it is ensured that all the chemical and physical properties (such as concentration, pH, temperature, shear force, etc.) remain constant. Due to the uniform reaction conditions, high-quality products can be obtained in both single-step and multi-step reactions (Shestopalov et al., 2004[106]; Chokkalingam et al., 2010[15]). Advances in microfluidic technology have revolutionized molecular biology methods for enzy[15]matic analysis (glucose and lactate assays), DNA analysis (high-throughput polymerase chain reaction and high-throughput sequencing), and proteomics. The main idea behind physiological devices is to integrate evaluation operations such as diagnosis, sample pre-testing, and sample provision on a single chip (Herold and Rasooly, 2009[42][43]). Microfluidic technology is a potent tool for researchers to control cells’ environment surrounding, as well as to answer new questions and make new discoveries. In the following, a number of advantages of this technology for biological sciences are listed:

- General studies of single cell including cell growth and proliferation (Manbachi et al., 2008[70]; Wang et al., 2010[133])
- Cell senescence: microfluidic devices such as "mother machine" allow for following thousands of generations of cells from birth to death (Manbachi et al., 2008[70])
- Control of microenvironment from mechanical to chemical environments (Chung et al., 2007[16]; Yliperttula et al., 2008[147])
- Creating precise spatio-temporal concentration gradients by combining the input of several chemicals into one device (Pelletier et al., 2012[86])
- Measuring adherent cell forces or chromosomes: microfluidic objects can be trapped using optical instruments or other means of energy production (Amir et al., 2014[2])
- Restricting cells and exerting controlled forces by two outer force-generating techniques such as stokes flow, optical tweezers, or Polydimethylsiloxane (PDMS)-controlled deformation (Choi et al., 2010[14]; Zhang et al., 2010[150]; Amir et al., 2014[2]; Houssin et al., 2016[45]; Li et al., 2016[66])
- Quick and precise temperature control (Yetisen et al., 2011[146]; Houssin et al., 2016[45])
- Electric field integration (Myers et al., 2011[76])
- Planting on a chip and planting tissue culture (Chang and Yeo, 2010[10])
- Antibiotic resistance: microfluidic devices can be applied as heterogeneous environments for microorganisms. In a heterogeneous environment, microorganisms can evolve easier. This capacity of microfluidic devices is useful for testing the growth rate of microorganisms or testing antibiotic resistance.

## Microfluidics and Tissue Engineering

Microfluidic technology can be applied to manipulate fluids at microliter to picoliter levels in specific environments, devices, and structures (Whitesides, 2006[137]; Karimi et al., 2016[51]). The combination of microfluidic systems and stem cells can create favorable conditions for stem cell culture, resulting in simultaneous proliferation of cells with uniform populations in a 3D environment (van Duinen et al., 2015[131]).

## Microfluidics and Isolation of Stem Cells

Stem cells environment is influenced by many physical and chemical factors such as calcium ions, different growth factors, nutrients, and oxygen. All these factors can affect cellular interactions. They affect cell-to-cell or cell-to-matrix interactions. Stem cell behavior is affected by these types of interferences; for example soft matrix enhances neural differentiation or stiff matrix induces myogenic and osteogenic differentiation (Gupta et al., 2010[36]). Microfluidics allows the exertion of precise control on the number of stem cells and their growth conditions. Microfluidics with pneumatic valves, osmotic pumps, and gradient-based production for nutrient gradient studies enables researchers to control 3D environments. Moreover, it also provides cells with the ability of tracking responses to a variety of mechanical, chemical, and optical stimuli (El-Ali et al., 2006[26]; Halldorsson et al., 2015[38]). This technology is also capable of precisely controlling mechanical factors including biomolecular stresses in the cell. Each stimulus has different effect on the fate of stem cells. In microfluidics, stem cells can be cultured in a 3D environment controlled by stresses resulted from the alterations in oxygen, pH, temperature, and nutrients (Lucchetta et al., 2005[68]). Microfluidic devices have an automatic fluorescence surface and good transparency to facilitate cell imaging. Many benefits have been mentioned for the use of PDMS in microfluidics in the field of stem cells biology. For example, PDMS-based systems are used to differentiate adipose-derived stem cells in the field of nerve tissue engineering (Choi et al., 2011[13]; Yang et al., 2013[143], 2015[144]; Kang et al., 2014[50]). Micro-divisions are based on natural polymers using gelatin agarose and collagen to simulate *in vivo* (Tsugita et al., 2000[128]; Park et al., 2009[85]; Lee et al., 2015[64]). Microfluidics can also be used to simultaneously study stem cell properties like differentiation and proliferation in contact with several stimuli of different origins (Park et al., 2009[85]). For example, in one study on neural stem cell tissue engineering, two sets of Embryonic Stem Cells (ESCs) and NSCs were used and researchers applied microfluidics to simultaneously culture different neurons such as glial cells, astrocytes, and Schwann cells, as well as to examine the effect of different stimuli on cellular properties (Harink et al., 2013[40]).

One of the most important sources for the separation of stem cells is ICM or blastocyst. The development of IPS cells, which produce all differentiated cell types including nerve cells, is one of the major stem cell-based research topics. The development of IPS cells can be achieved by differentiating somatic stem cells under specific conditions. IPS cells can produce all differentiated cell types such as nerve cells (Eiraku and Sasai, 2012[25]). Microfluidics can create good conditions for the differentiation pathway of these neurons which can be applied to treat a variety of neurological diseases including genetic disorders. Here, cell culture is conducted in two ways: gel-based and gel-free approaches (Choi et al., 2011[13]). Each has its own pros and cons (Zhou et al., 2012[151]; Shin et al., 2014[110]; Cosson and Lutolf, 2015[17]). In the gel-free method, stem cell cultures are used for long-term, while the gel-based method has good cause to be similar biomass *in vivo* environment (Bond et al., 2012[5]).

In recent years, many studies have been conducted on using microfluidic platforms in the field of neurobiology research (Park et al., 2009[84]; Taylor and Jeon, 2011[122]; Yamada et al., 2016[142]). Microfluidic devices make the observation of different types of neuronal differentiation possible (axon and cell body), that greatly helps to study neurodegenerative diseases. In this context, exons traverse the microfluidic length and eventually separate from the somatic cell body. This application of microfluidics helps in exploring the biology of axons (Shin et al., 2010[109]). In addition, utilizing microfluidics enables researchers to screen ESCs that are removed from blastocyst in the early embryonic stages and examine their proliferation and differentiation (Thomson, 1998[125]; Desbaillets et al., 2000[19]; Khademhosseini et al., 2006[53]; Samadikuchaksaraei et al., 2006[102]). During differentiation, ESCs produce bodies called Embryoid bodies (Jastrzebska et al., 2016[48]), the 3D cells formed by culturing ESCs in an uncoordinated substrate. EBs can be examined in microfluidics by determining the number of clusters. Cluster differentiation is difficult to control in large-scale systems. Thus, microfluidics are efficient to produce uniform EBs with adjustable sizes. This technique provides the generation of uniform ESCs in a particular area (Torisawa et al., 2007[127]; Nguyen et al., 2009[79]; Wu et al., 2011[140]; Edalat et al., 2012[24]). In general, microfluidic systems, both physical and chemical properties, can be studied and mechanical forces play a key role in stem cell differentiation and behavior. It has been shown that cell colonies with healthy morphology have a high growth rate and microfluidic systems can be considered as a good option for the study of cells under these conditions (Table 1(Tab. 1); References in Table 1: Gothard et al., 2011[33]; Green and Murthy, 2009[34]; Hatch et al., 2012[41]; LV et al., 2012[69]; Ng et al., 2010[78]; Pertoft, 2000[87]; Pethig, 2010[88]; Pruszak et al., 2007[94]; Roda et al., 2009[99]; Slámová, 2014[111]; Smith et al., 2012[114]; Srisa-Art et al., 2009[116]; Stephens et al., 1996[117]; Wang et al., 2000[134]; Will and Steidl, 2010[138]; Wu and Morrow, 2012[139]).

## Perspectives

In recent years, many strategies have been applied to differentiate and cultivate stem cells in microfluidic systems, but there are still challenges to be solved over time. One of the main challenges in using microfluidics for stem cells is that it takes hours, with existing devices, to obtain several milliliters of stem cell samples however, using multichannel arrays can result in achieving higher efficiency in a short time period.

## Conclusion

Microfluidic systems provide a very small-scale environment for stem cells which is similar to the body environment. The main advantage of these systems is their small size, low sample, and reagent consumption. Microfluidic platforms are also capable of performing several test steps with increased efficiency and high speed within short time intervals. Moreover, high optical transparency of these systems provides accurate and instantaneous imaging and analysis of cellular responses to different stimuli. However, the commercial production of microfluidic devices for stem cell research faced with some challenges. One of the biggest problems is that microfluidics have been used extensively in cancer therapy rather than stem cell research. The reason is that cancer accounts for a large percentage of clinical problems in human societies. However, microfluidics is very efficient to investigate the effects of drugs on cell performance. The viability of stem cells can be explored by evaluating the effect of different parameters on the metabolic rate of cells.

## Notes

Leili Aghebati-Maleki and Mehdi Yousefi (Stem Cell Research Center, Tabriz University of Medical Sciences, Tabriz, Iran; Tel: +98-4133364665, Fax: +98-4133364665, E-mail: yousefime@tbzmed.ac.ir) contributed equally as corresponding authors.

## Conflict of interest

The authors declare no conflict of interest.

## Funding

None.

## Acknowledgement

None.

## Authors’ contribution

Afsoon Aghlmandi wrote the article. Aylin Nikshad and Reza Safaralizadeh wrote the initial draft of the manuscript and prepared figures. Majid Ebrahimi Warkiani and Leili Aghebati-Maleki reviewed and edited the final version of the manuscript. Mehdi Yousefi supervised the study.

## Data availability statement

The data that support the findings of this study are available from the corresponding author upon reasonable request.

## Figures and Tables

**Table 1:**
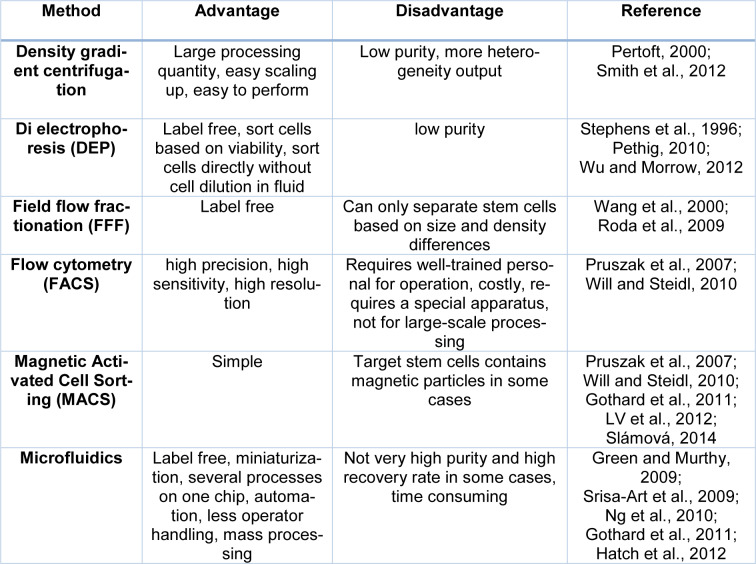
Advantages and disadvantages of Stem Cell Separation Technologies
